# Association between NDRG1 protein expression and aggressive features of breast cancer: a systematic review and meta-analysis

**DOI:** 10.1186/s12885-023-11517-7

**Published:** 2023-10-19

**Authors:** Kwuntida Kotepui, Manas Kotepui, Hideyuki J. Majima, Jitbanjong Tangpong

**Affiliations:** Medical Technology, School of Allied Health Sciences, Walailak University, Tha Sala, Nakhon Si Thammarat, Thailand

**Keywords:** NDRG1, Breast cancer, Aggressiveness, Metastasis

## Abstract

**Background:**

N-myc downstream-regulated gene-1 (NDRG1) is well-described as a potent metastasis suppressor, but its role in human breast cancer remains controversial and unclear. Therefore, the present study utilized a systematic review and meta-analysis approach to synthesize the association between NDRG1 protein expression and the aggressive characteristics of breast cancer.

**Methods:**

The protocol for the systematic review and meta-analysis was registered on the PROSPERO website (CRD42023414814). Relevant articles were searched for in PubMed, Scopus, Embase, MEDLINE, and Ovid between March 30, 2023, and May 5, 2023. The included studies were critically evaluated using the Joanna Briggs Institute critical appraisal tools. The results from individual studies were qualitatively synthesized using textual narrative synthesis. Using a random-effects model, the pooled log odds ratio of effect estimate was used to look at the link between NDRG1 protein expression and aggressive features of breast cancer, such as tumor grade, tumor stage, metastasis to the axillary lymph nodes, and hormonal receptor status.

**Results:**

A total of 1423 articles were retrieved from the electronic database search, and six studies that met the eligibility criteria were included for synthesis. There was an association between the expression of NDRG1 protein and the status of the axillary lymph nodes (*P* = 0.01, log Odds Ratio (OR): 0.59, 95% Confidence Interval (CI): 0.13–1.05, I^2^: 24.24%, 292 breast cancer cases with positive axillary lymph nodes and 229 breast cancer cases with negative axillary lymph nodes, 4 studies). NDRG1 protein expression and human epidermal growth factor receptor 2 (Her2) status were found to have a negative relationship (*P* = 0.01, log OR: -0.76, 95% CI: -1.32–(-0.20), I^2^: 32.42%, 197 breast cancer cases with Her2 positive and 272 breast cancer cases with Her2 negative, 3 studies). No correlation was found between NDRG1 protein expression and tumor grade (*P* = 0.10), estrogen receptor (ER) status (*P* = 0.57), or progesterone receptor (PR) status (*P* = 0.41).

**Conclusion:**

The study concluded that increased NDRG1 protein expression was associated with increased metastasis of the tumor to the axillary lymph node. Additionally, increased NDRG1 protein expression was observed in Her2-negative breast cancer, suggesting its role in both less aggressive and more aggressive behavior depending on breast cancer subtypes. Based on the findings of the meta-analysis, an increase in NDRG1 protein expression was associated with aggressive characteristics of breast cancer.

**Supplementary Information:**

The online version contains supplementary material available at 10.1186/s12885-023-11517-7.

## Introduction

In 2020, there were over 2 million new cases of breast cancer, making it the fifth leading cause of cancer-related mortality and the most frequently diagnosed malignancy in women [1, 2]. Furthermore, the death rates of breast cancer have continuously increased, particularly in developing countries compared to those that have already transitioned [2]. Breast cancer is caused by various risk factors, including genetic and hereditary predispositions [3]. Approximately 30% of breast cancer cases are linked to obesity, physical inactivity, and alcohol consumption [4]. Factors associated with poor clinical outcomes or shorter survival in breast cancer include tumor size, tumor grade, axillary lymph node status, estrogen receptor (ER) status, progesterone receptor (PR) status, and human epidermal growth factor receptor 2 (Her2/neu) status [5, 6].

N-myc downstream-regulated gene-1 (NDRG1) is found on the human chromosome 8q24.3. It is part of the new NDRG family, which is part of the alpha/beta hydrolase superfamily [7]. NDRG1 is predominantly cytoplasmic but can also be found in the cellular membrane and adherens junctions [8]. It has gained recognition as a potent metastasis suppressor by inhibiting epithelial-mesenchymal transition (EMT), cell migration, and angiogenesis [9]. Several targets of NDRG1-related antitumor function have been identified, including the suppression of activating transcription factor 3 (ATF3) expression [10], cathepsin C [11], and I kappa B kinase (IKK) expression, as well as I kappa B alpha (IKBα) phosphorylation [12]. NDRG1 also enhances the expression of thiamine triphosphatase (Thtpa) [11], and E-cadherin, attenuating the oncogenic role of TGF-β and NF-κB signaling [13]. Kovacevic et al. suggested that the antitumor function of NDRG1 is mediated by inducing the expression of the potent cyclin-dependent kinase inhibitor, p21, through p53-independent mechanisms [14].

Although the postulated function of NDRG1 as a suppressor of breast cancer metastasis and other cancers exists, the relevance of NDRG1 in human breast cancer remains uncertain and speculative. The most recent and comprehensive review by Zhao and Richardson suggests that the role of NDRG1 may be independent of breast cancer subtypes [15]. Furthermore, the results of individual studies were based on a small number of tissue samples, which could contribute to statistical bias in reporting the association. Therefore, the present study adopts a systematic review and meta-analysis approach to synthesize the evidence and investigate the association between NDRG1 expression and the aggressive characteristics of breast cancer.

## Methods

### Registration of the protocol and guidelines for reporting

The systematic review and meta-analysis protocol was registered on the PROSPERO website (CRD42023414814). The reports of the systematic review followed the PRISMA 2020 statement [16].

### Searches of literature

The search for relevant articles will be performed in PubMed, Scopus, Embase, MEDLINE, and Ovid. The following search strategy had been applied in the search databases: “(NDRG1 OR RIT42 OR RIT42 OR “nickel-specific induction protein Cap43” OR “N-myc downstream regulated gene 1 protein” OR “N-myc downstream-regulated gene 1” OR “differentiation-related gene 1”) AND (Breast OR Mammary) AND (Neoplasm* OR Neoplasia* OR Tumor* OR Cancer* OR Carcinoma* OR Malignan*). The reference lists of the selected studies were additionally searched to ensure that all relevant articles were not missed. The searches began on March 30, 2023, and ended on May 5, 2023, without limitation of language or publication year.

### Eligibility criteria

The following studies were included in the study: (i) studies that reported NDRG1 protein expression in breast cancer tissues (clinical samples) by immunohistochemistry or tissue array methods; and (ii) studies that reported NDRG1 protein expression concerning tumor size, tumor grade, stage, axially lymph node status, ER status, PR status, and Her2 status. The following studies were excluded from the study: (i) studies that investigated NDRG1 protein expression in breast cancer tissues after patients underwent treatment; (ii) conference abstracts without full text; and (iii) non-original articles such as case reports or case studies, NDRG1 gene expression (mRNA), in vitro studies, in vivo studies, reviews, and systematic reviews.

### Study selection and data extraction

After articles were retrieved from database searches, the articles from each database were imported into Endnote 20.0 software for reference management (Clarivate, Philadelphia, PA). The article titles and abstracts were reviewed for potentially related articles, and the relevant studies were examined for full texts against the eligibility criteria. Data on the first author (and year of publication), country (and continent), study design, participant demographics (age, gender), tumor grading, stage of cancer, NDRG1 protein expression (qualitative, semi-quantitative), ER status, PR status, and Her2 status were extracted into a Excel spreadsheet (Microsoft Corporation, USA) for analysis. The selection and extraction of the study were performed independently by two authors (KUK and MK). Any discrepancy in study selection and data extraction between the two authors was settled through discussion.

### Risk of bias

The eligible studies were critically appraised for the risk of bias using the Joanna Briggs Institute (JBI) critical appraisal tools for observational studies [17]. There were eight checklist items for cross-sectional studies and 11 checklist items for cohort studies. The risk of bias was performed independently by two authors (KUK and MK). Any discrepancy in the risk of bias assessment between the two authors was resolved by discussion to create consensus.

### Data syntheses

There were two steps to data synthesis. First, the results from individual studies were synthesized qualitatively using textual narrative synthesis, which is the method for synthesizing the outcomes from homogenous groups of studies [18]. Second, the quantitative synthesis of the meta-analysis was performed. The significance and levels of heterogeneity of outcome between studies were assessed using Chi-square (Q) and I^2^ statistics, respectively. The pooled log odds ratio of the effect estimate was computed using a random-effects model as described by DerSimonian and Laird in case the estimate estimates from studies were heterogeneous (*P* value for Q statistic < 0.10 or I^2^ > 50% [19]. If the effect estimates from studies were homogenous (*P* value for Q statistic > 0.10 or I^2^ < 50%), a fixed-effects model was used to pool the log odds ratio of the effect estimate. The subset meta-analyses were as follows: (i) association between NDRG1 protein expression and tumor grading (grade III vs. I + II), (ii) association between NDRG1 protein expression and tumor stage (stage III + IV vs. I + II), (iii) association between NDRG1 protein expression and axillary lymph node metastasis (metastasis vs. non-metastasis); and (iv) association between NDRG1 protein expression and ER, PR, and Her2 status (positive receptor vs. negative receptor). Meta-regression and analyses of subgroups were used to investigate the potential source(s) of outcome heterogeneity across studies. The publication bias and small-study effect were assessed by visualization of the funnel plot symmetry, Egger’s test, and a contoured-enhanced funnel plot if more than 10 studies were included in the meta-analysis [20]. Stata software version 17.0 (Stata Corp. College Station, TX) was used to perform the meta-analysis.

## Results

### Search results

**Fig. 1:**
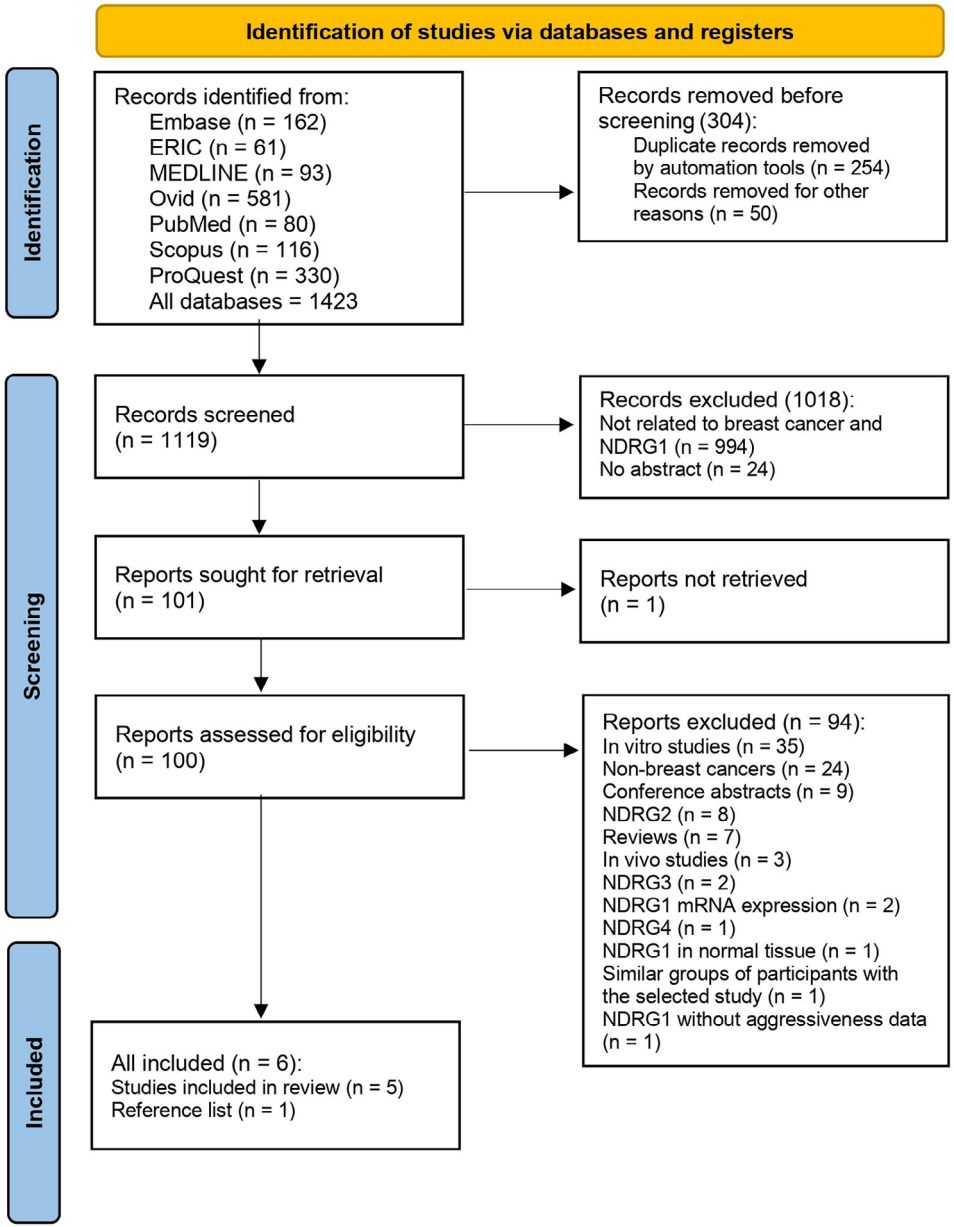
Flow diagram of study selection

A total of 1423 articles were retrieved from searching Embase (n = 162), ERIC (n = 61), MEDLINE (n = 93), Ovid (n = 581), PubMed (n = 80), Scopus (n = 116), and ProQuest (n = 330). After 304 duplicates were removed, 1119 articles underwent screening. Among them, 100 were further examined for full text according to the eligible criteria. Finally, six studies [21–26] met the eligible criteria were included for syntheses (Fig. 1).

### Characteristics and risk of bias of the studies

Studies were published between 2011 and 2023 (Table 1). Four studies were cohort [21, 22, 24, 26], and two were cross-sectional studies [23, 25]. Four studies enrolled patients with invasive breast cancers [22, 23, 25, 26]. Meanwhile, other studies enrolled patients with inflammatory breast cancer [24] and only triple-negative breast cancer [21]. Four studies used immunohistochemistry for detection of NDRG1 protein expression [21, 22, 24, 26]. Meanwhile, another study used only tissue microarrays [25], and another study used both tissue microarrays and immunohistochemistry methods for the detection of NDRG1 protein expression [23]. The results of the critical appraisal for the risk of bias in observational studies are shown in Table S2.

**Table 1:** Studies ‘characteristics

No.	Name of the first author	Publication year	Study design	Year of the study	Participants	Number of participants with breast cancer	Age range	Qualitative data of NDRG1 in relation to aggressive breast cancer	NDRG1 cutoff score
1	López-Tejada et al.	2023	Cohort study	Not specified	Triple-negative breast cancer	83	Not specified	High expression of NDRG1 was associated with poorer cumulative patient survival (mean survival: 6.9 years) compared to a lower expression (mean survival: 15.1 years)	199
2	Mao et al.	2011	Cohort study	2007–2011	Breast normal tissues (n = 35), pure usual ductal hyperplasia (UDH, n = 40), atypical ductal hyperplasia (ADH, n = 22), atypical lobular hyperplasia (ALH, n = 8), ductal carcinoma in situ (DCIS, n = 16), lobular carcinoma in situ (LCIS, n = 6), invasive ductal carcinoma (IDC, n = 50), and invasive lobular carcinoma (ILC, n = 45)	95	15–69	NDRG1 expression not associated with tumor size, and axillary lymph node metastasis, but it was significantly associated with tumor stage	≥ 2 final score
3	Nagai et al.	2011	Cross-sectional study	Not specified	Invasive breast cancer (ductal carcinoma)	754	24–96	Positive NDRG1 protein expression was significantly associated with tumor size, tumor grade, axillary lymph node metastasis, tumor stage, ER, and PR status	80
4	Villodre et al.	2020	Cohort study	1991–2004	Inflammatory breast cancer	NDRG1-Low (n = 32), NDRG1-High (n = 32)	23–75	NDRG1 expression was significantly associated with Her2 status but not associated with tumor grade, stage, axillary lymph node metastasis, ER, PR, and triple-negative breast cancer	120
5	Villodre et al.	2022	Cross-sectional study	Not specified	Invasive breast cancer (not specified)	NDRG1-high (n = 101), NDRG1-low (n = 115)	Not specified	NDRG1 expression was significantly associated with tumor grade, ER, PR, and triple-negative breast cancer but not associated with Her2 status	≤ median score
6	Zeng et al.	2019	Cohort study	1996–2005	Invasive breast cancer (ductal and lobular carcinoma)	190	Not specified	NDRG1 expression was significantly associated with tumor grade, but not associated with tumor size, axillary lymph node metastasis, ER, PR, and Her2 status	Not specified

### Qualitative synthesis

**Fig. 2:**
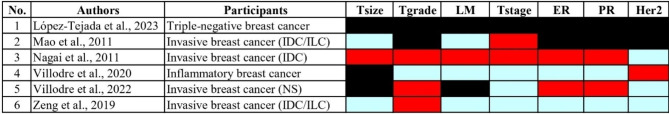
Results of an individual study investigating NDRG1 protein expression concerning aggressive breast cancer. **Abbreviation**: *IDC*, invasive ductal carcinoma; *ILC*, Iinvasive lobular carcinoma; *NS*, not specified subtypes; *Tsize*, tumor size; *Tgrade*, tumor grade; *LM*, lymph node metastasis; *ER*, estrogen receptor; *PR*, progesterone receptor; *Her2*, human epidermal growth factor receptor 2. *Black-colored box*, not investigate or not specify the association; *Red-colored box*, significantly associated with NDRG1 protein expression; *Blue-colored box*, no association with NDRG1 protein expression

Results of individual studies investigating NDRG1 protein expression concerning aggressive breast cancer are illustrated in Fig. 2. López-Tejada et al. demonstrated that high NDRG1 protein expression was related to shorter patient survival (6.9 years vs. 15.1 years) [21]. Nagai et al. demonstrated that the expression of the NDRG1 protein was significantly associated with tumor grade [23]. The expression of the NDRG1 protein was significantly associated with tumor grade, as shown by Nagai et al. [23], Villodre et al. [25], and Zeng et al. [26]. Nagai et al. demonstrated that NDRG1 protein was significantly associated with lymph node metastasis [23]. Mao et al. [22] and Nagai et al. [23] demonstrated a significant relationship between NDRG1 protein expression and tumor stage. According to Nagai et al. [23] and Villodre et al. [25], the expression of the NDRG1 protein was substantially correlated with ER status. According to Nagai et al. [23] and Villodre et al. [25], the expression of the NDRG1 protein was significantly correlated with PR status. Villodre et al. [24] demonstrated that NDRG1 protein expression was significantly related to Her2 status.

### Association between NDRG1 protein expression and tumor grading

**Fig. 3:**
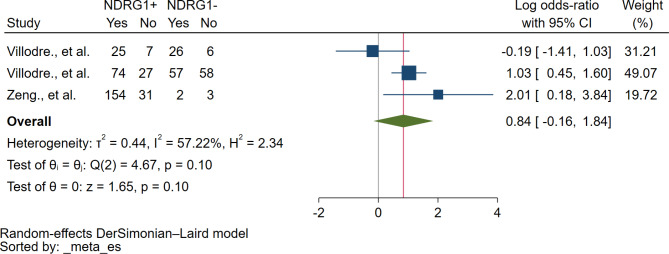
The forest plot demonstrates the association between NDRG1 protein and tumor grading. **Abbreviation**: *NDRG1+*, NDRG1 expression was positive or increased; *NDRG1-*, NDRG1 expression was negative or decreased; *Yes*, breast cancer with tumor grade III; *No*, breast cancer tumor grades I + II; *Blue squared-box*, point estimate; *Blue horizontal line*, 95% confidence interval; *Green diamond*, pooled log OR; *Red vertical line*, pooled log OR; *Brawn vertical line*, no effect line

Based on the meta-analysis of three studies, no correlation between NDRG1 protein expression and tumor grading was identified (*P* = 0.10, log OR: 0.84, 95% CI: 0.16–1.84, I^2^: 57.22%, 338 breast cancer with tumor grade III/132 breast cancer with tumor grade I + II, 3 studies, Fig. 3).

### Association between NDRG1 protein expression and tumor stage

**Fig. 4:**
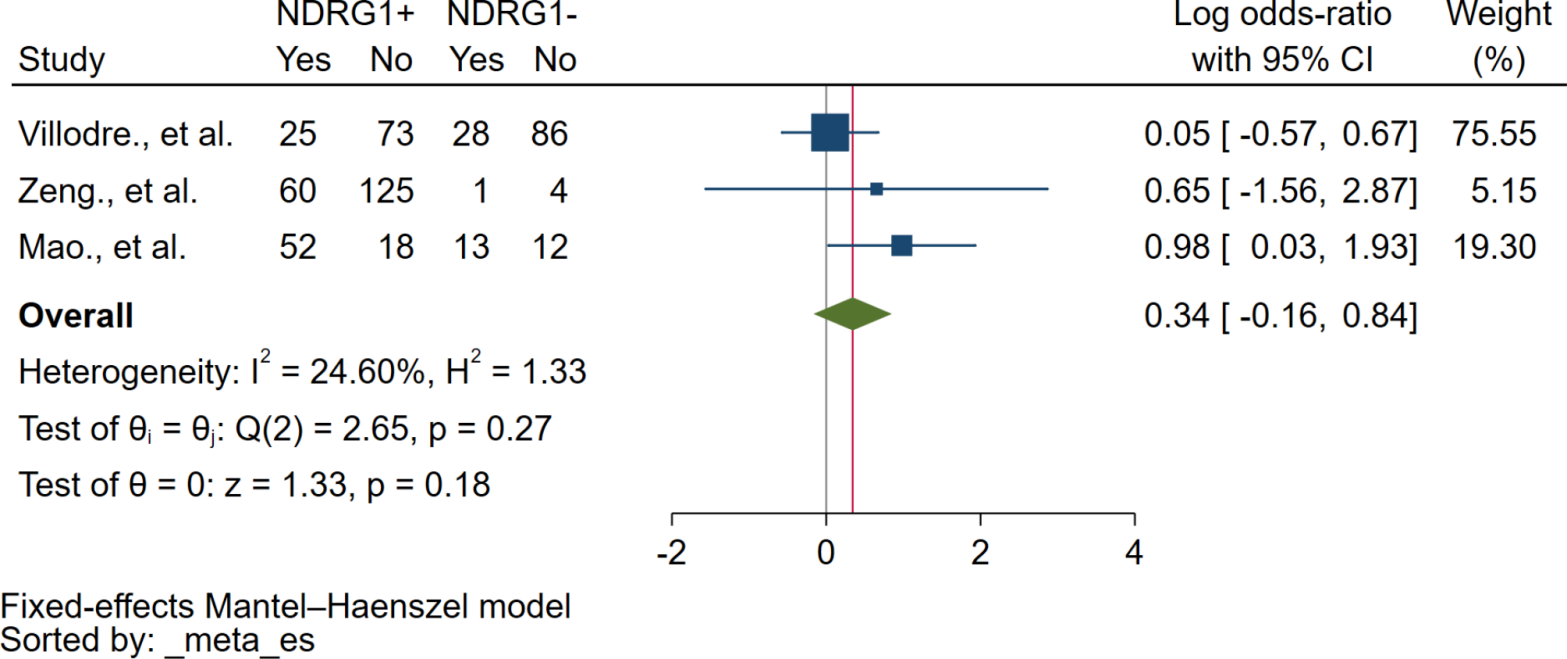
The forest plot demonstrates the association between NDRG1 protein and tumor stage. **Abbreviation**: *NDRG1+*, NDRG1 expression was positive or increased; *NDRG1-*, NDRG1 expression was negative or decreased; *Yes*, breast cancer with tumor stage III/IV; *No*, breast cancer with tumor stage I + II; *Blue squared-box*, point estimate; *Blue horizontal line*, 95% confidence interval; *Green diamond*, pooled log OR; *Red vertical line*, pooled log OR; *Brawn vertical line*, no effect line

Based on the meta-analysis of three studies, no correlation between NDRG1 protein expression and tumor stage was identified (*P* = 0.18, log OR: 0.34, 95% CI: -0.16-0.84, I^2^: 24.60%, 179 breast cancer with tumor stage III + IV/318 breast cancer with tumor stage I + II, 3 studies, Fig. 4).

### Association between NDRG1 protein expression and axillary lymph node status

**Fig. 5:**
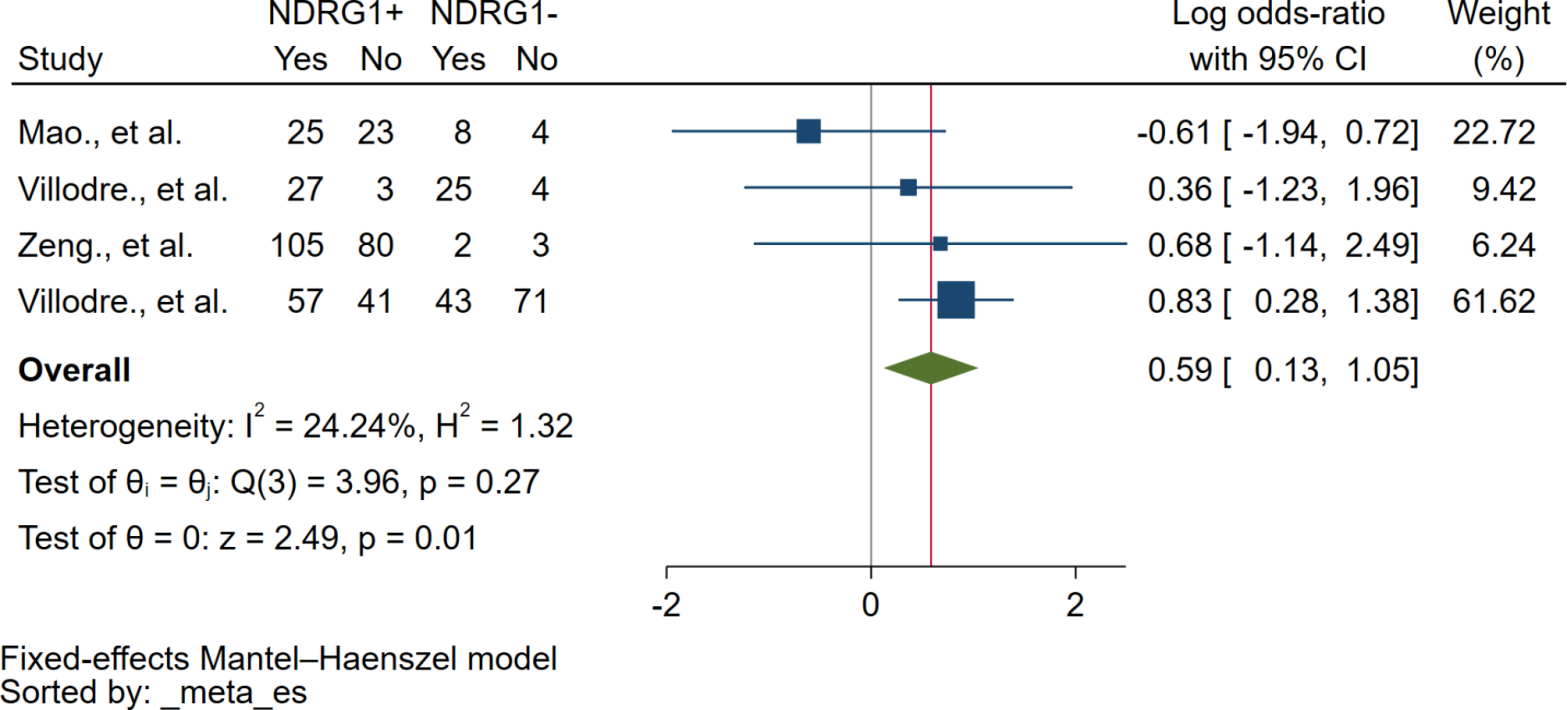
The forest plot demonstrates the association between NDRG1 protein and lymphatic metastasis in breast cancer. **Abbreviation**: *NDRG1+*, NDRG1 expression was positive or increased; *NDRG1-*, NDRG1 expression was negative or decreased; *Yes*, breast cancer with axillary lymph node metastasis; *No*, breast cancer without axillary lymph node metastasis; *Blue squared-box*, point estimate; *Blue horizontal line*, 95% confidence interval; *Green diamond*, pooled log OR; *Red vertical line*, pooled log OR; *Brawn vertical line*, no effect line

Based on the meta-analysis of three studies, there was a positive correlation between NDRG1 protein expression and axillary lymph node metastasis (*P* = 0.01, log OR: 0.59, 95% CI: 0.13–1.05, I^2^: 24.24%, 292 breast cancer with axillary lymph node-positive/229 breast cancer with axillary lymph node-negative, 4 studies, Fig. 5).

### Association between NDRG1 protein expression and ER status

**Fig. 6:**
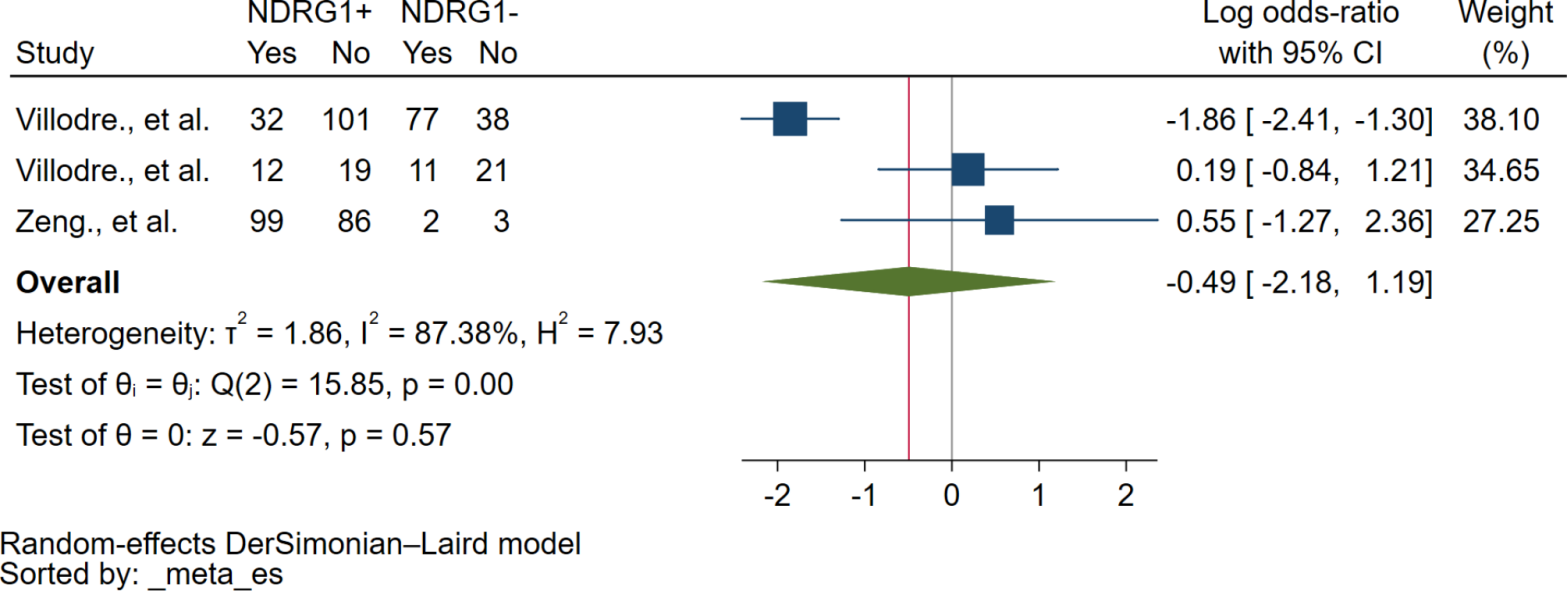
The forest plot demonstrates the association between NDRG1 protein and ER status. **Abbreviation**: *NDRG1+*, NDRG1 expression was positive or increased; *NDRG1-*, NDRG1 expression was negative or decreased; *Yes*, breast cancer with ER+; *No*, breast cancer with ER-; *Blue squared-box*, point estimate; *Blue horizontal line*, 95% confidence interval; *Green diamond*, pooled log OR; *Red vertical line*, pooled log OR; *Brawn vertical line*, no effect line

Based on the meta-analysis of three studies, no correlation between NDRG1 protein expression and ER status was identified (*P* = 0.57, log OR: -0.49, 95% CI: -2.18-1.19, I^2^: 87.38%, 233 breast cancer with ER-positive/268 breast cancer with ER-negative, 3 studies, Fig. 6).

### Association between NDRG1 protein expression and PR status

**Fig. 7:**
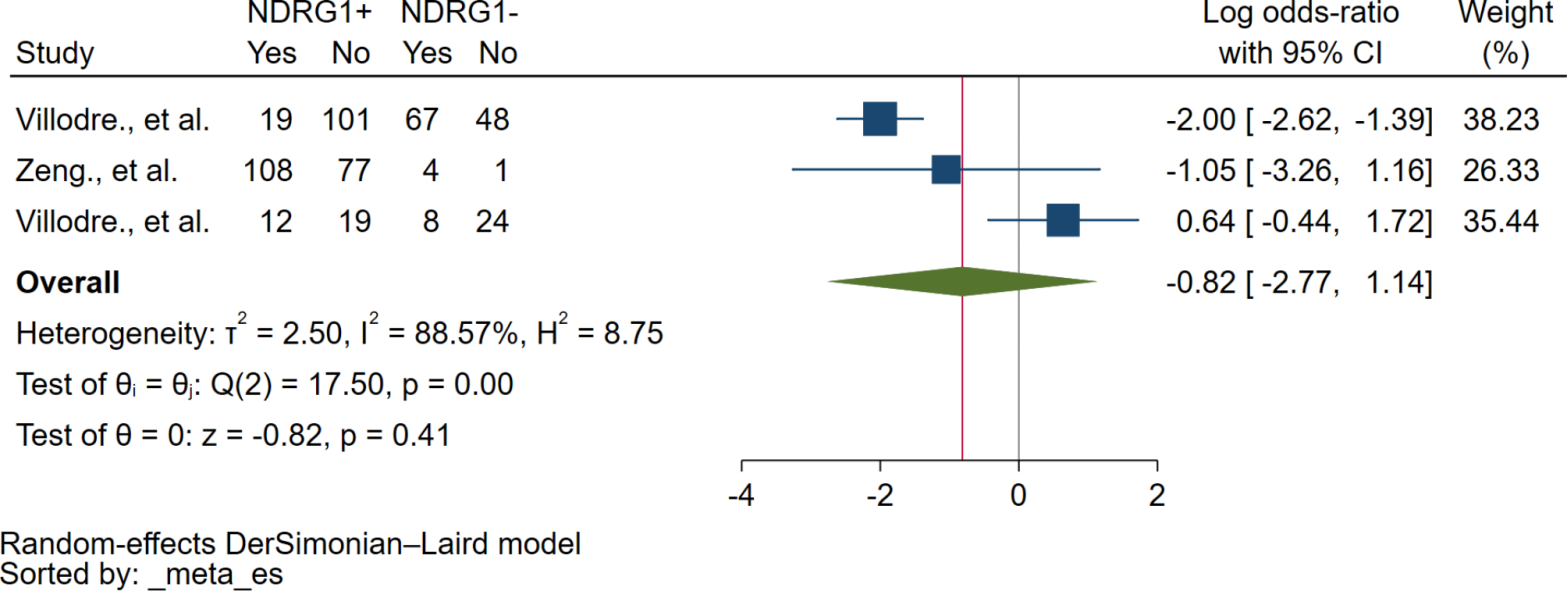
The forest plot demonstrates the association between NDRG1 protein and ER status. **Abbreviation**: *NDRG1+*, NDRG1 expression was positive or increased; *NDRG1-*, NDRG1 expression was negative or decreased; *Yes*, breast cancer with PR+; *No*, breast cancer with PR-; *Blue squared-box*, point estimate; *Blue horizontal line*, 95% confidence interval; *Green diamond*, pooled log OR; *Red vertical line*, pooled log OR; *Brawn vertical line*, no effect line

Based on the meta-analysis of three studies, no correlation between NDRG1 protein expression and PR status was identified (*P* = 0.41, log OR: -0.82, 95% CI: -2.77-1.14, I^2^: 88.57%, 218 breast cancer with PR positive/270 breast cancer with PR negative, 3 studies, Fig. 7).

### Association between NDRG1 expression and Her2 status

**Fig. 8:**
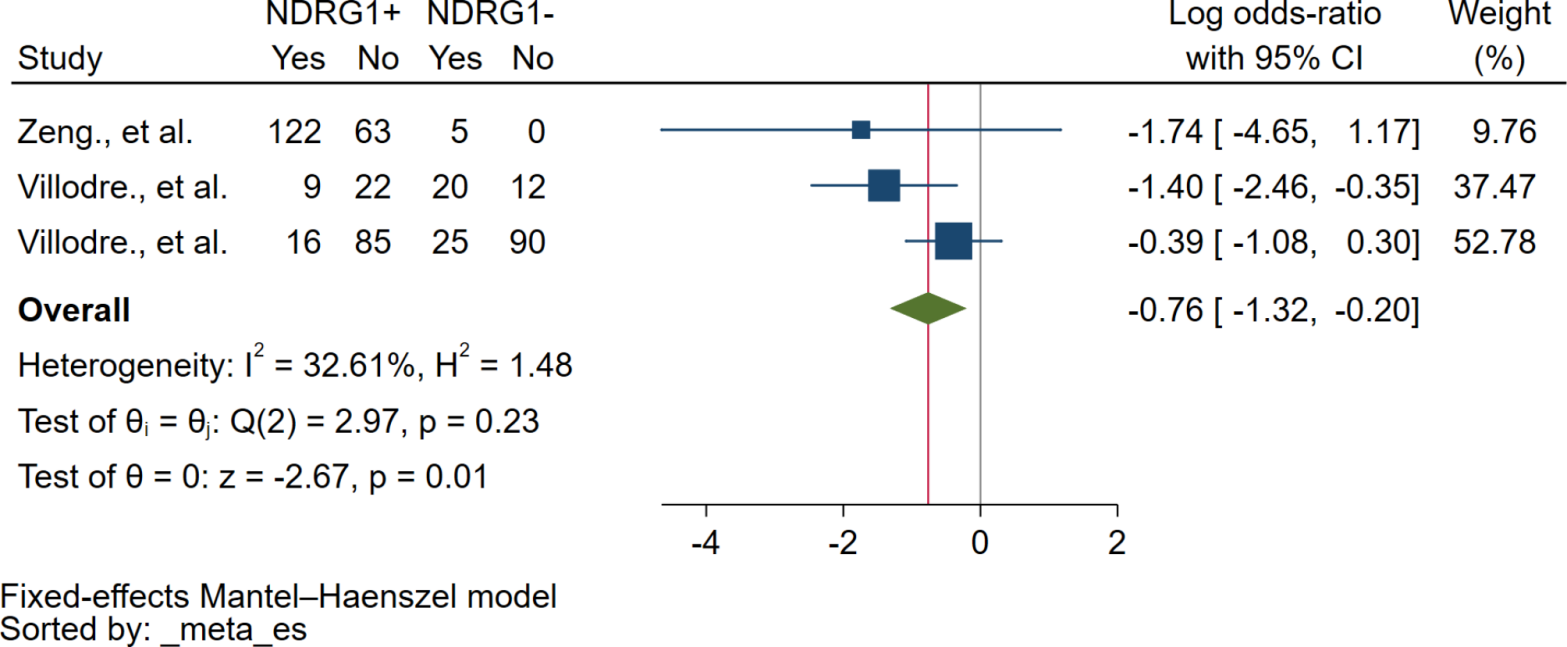
The forest plot demonstrates the association between NDRG1 protein and ER status. **Abbreviation**: *NDRG1+*, NDRG1 expression was positive or increased; *NDRG1-*, NDRG1 expression was negative or decreased; *Yes*, breast cancer with Her2+; *No*, breast cancer with Her2-; *Blue squared-box*, point estimate; *Blue horizontal line*, 95% confidence interval; *Green diamond*, pooled log OR; *Red vertical line*, pooled log OR; *Brawn vertical line*, no effect line

Based on the meta-analysis of three studies, there was a negative correlation between NDRG1 protein expression and Her2 status (*P* = 0.01, log OR: -0.76, 95% CI: -1.32-(-0.20), I^2^: 32.42%, 197 breast cancer with Her2 positive/272 breast cancer with Her2 negative, 3 studies, Fig. 8).

### Sensitivity analysis

The association between NDRG1 protein expression and aggressive features of breast cancer was analyzed using different statistical models based on the significance of the Q or I^2^ statistics, as mentioned in the method section. After the statistical model was changed in the meta-analysis, results showed a positive correlation between NDRG1 protein expression and tumor grading according to the fixed-effects model (*P* < 0.01, log OR: 0.87, 95% CI: 0.38–1.37, I^2^: 57.29%, 3 studies, Supplementary Fig. 1). There was no correlation between NDRG1 protein expression and tumor stage was identified according to the random effects model (*P* = 0.21, log OR: 0.42, 95% CI: -0.24-1.07, I^2^: 24.59%, 3 studies, Supplementary Fig. 2). No correlation between NDRG1 protein expression and axillary lymph node metastasis according to the random effects model (*P* = 0.16, log OR: 0.47, 95% CI: -0.18-1.12, I^2^: 24.16%, 4 studies, Supplementary Fig. 3). There was a negative correlation between NDRG1 protein expression and ER status according to the random effects model (*P* < 0.01, log OR: -1.22, 95% CI: -1.67-(-0.77), I^2^: 87.40%, 3 studies, Supplementary Fig. 4). There was a negative correlation between NDRG1 protein expression and PR status according to the fixed-effects model (*P* < 0.01, log OR: -1.29, 95% CI: -1.78-(-0.18), I^2^: 88.57%, 3 studies, Supplementary Fig. 5). There was a negative correlation between NDRG1 protein expression and Her2 status according to the random effects model (*P* = 0.04, log OR: 0.86, 95% CI: -1.66-(-0.05), I^2^: 32.42%, 3 studies, Supplementary Fig. 6).

## Discussion

The association between NDRG1 protein expression and breast cancer was assessed by analyzing a larger number of samples from multiple publications. The results of the meta-analysis demonstrated a significant association between NDRG1 protein expression and the lymph node and Her2 statuses of breast cancer. However, no correlations were found between tumor grade, tumor stage, ER status, or PR status.

The meta-analysis results revealed a positive correlation between NDRG1 protein expression and lymph node status, suggesting that increased NDRG1 protein expression is associated with an increased spread of the tumor to the lymph nodes. These findings confirm previous research indicating that NDRG1 may play a crucial role in tumor development and metastasis, specifically in certain breast cancer subtypes [15]. NDRG1 exhibited a strong association with poor clinical outcomes and tumor characteristics associated with an aggressive phenotype. Villodre et al. [25] also reported positive correlations between NDRG1 expression and aggressive phenotype-associated tumor characteristics. Additionally, depletion of NDRG1 has been shown to reduce the invasion and migration of a specific breast cancer subpopulation [25]. The activation of mTOR-AKT signaling mediates the progression of NDRG1-related cancer [25]. However, previous studies have produced inconsistent results regarding the relationship between NDRG1 protein and breast cancer. Some studies have reported a significant direct association between NDRG1 and tumor grade, as well as axillary lymph node metastasis [23, 27]. In contrast, Mao et al. found no association between NDRG1 and tumor size or axillary lymph node metastasis, but they did find an association with tumor stage [22]. Another study by Villodre et al. [25] demonstrated that high NDRG1 expression was associated with tumor grade but not tumor stage.

The results of the meta-analysis showed a negative correlation between NDRG1 protein expression and Her2-negative breast cancer. This means that more NDRG1 protein expression is linked to a less aggressive type of breast cancer. This suggests that tumor cells with higher NDRG1 expression tend to grow slower and are less likely to spread [28]. Breast cancers that are hormone receptor (HR)-positive and Her2-negative are often diagnosed at an early stage, leading to improved survival outcomes [29]. However, HR+/ Her2- breast cancer has been reported to be associated with a risk of relapse or recurrence, highlighting the need for individualized treatment protocols and the determination of the optimal duration of adjuvant treatment [30, 31]. In contrast, the more aggressive type of breast cancer known as triple-negative breast cancer (TNBC), which lacks the expression of ER, PR, and Her2, has been reported to have a higher tendency for metastasis, a poorer prognosis, and higher relapse rates compared to non-TNBC [32]. Therefore, there exists a negative correlation between NDRG1 protein expression and TNBC, indicating that increased NDRG1 protein expression is associated with the aggressive features of breast cancer.

It has been proposed that NDRG1 exhibits pleiotropic actions depending on the type of tumor [9, 15]. Several studies have demonstrated that NDRG1 acts as a tumor and metastasis suppressor, exhibiting pleiotropic activity in various types of tumors, including colorectal cancer [33], lung cancer [34], esophageal squamous cell carcinoma [35], and breast cancer [36, 37]. In renal cell carcinoma, the suppression of *NDRG1* gene expression in vitro significantly increased renal cell proliferation and invasion [38]. Conversely, NDRG1 can stimulate tumor growth, spread, angiogenesis, and poor prognosis in other malignancies, such as lung cancer [34], bladder cancer [39], gastric cancer [40], and invasive breast cancer [24, 25]. In colorectal cancer, NDRG1 expression was suggested to transition from the membrane to the cell nucleus, which was associated with lymph node metastasis [33]. The biological mechanisms underlying the diverse actions of NDRG1 remain unknown, but different regulation of WNT signaling and possible differential interaction with the tumor suppressor PTEN are implicated [9]. A meta-analysis of NDRG1 expression using data from genomic databases revealed that an altered lipid metabolic phenotype in breast cancer cells contributes to the aggressiveness of the disease [37]. NDRG1 functions as a metastasis suppressor with diverse roles, inhibiting the spread of several cancers while also being associated with metastasis in certain tumors, depending on post-translational modifications such as NDRG1 phosphorylation and cleavage [41]. More recently, NDRG1 has emerged as a potential target for therapy and a predictive biomarker in aggressive breast cancers, as it significantly correlates with worse clinical outcomes [25].

The systematic review and meta-analysis had limitations. Firstly, the expression of the NDRG1 concerning breast cancer metastasis was investigated by researchers, and there were a limited number of studies that provided evidence of NDRG1 protein expression in patients with breast cancer. Therefore, the meta-analysis results were constrained by the scarcity of available studies included in the analysis. Secondly, in each conducted meta-analysis, the number of studies was less than or equal to four, which prevented the performance of a publication bias analysis. Further studies are crucially needed to investigate the expression of NDRG1 protein concerning breast cancer metastasis. Such studies can help establish the NDRG1 protein as an efficient biomarker to better characterize the prognosis of breast cancer.

## Conclusion

The systematic review and meta-analysis demonstrated that NDRG1 protein expression were associated with breast cancer progression. Elevated levels of NDRG1 protein were observed to have an association with the metastasis of the tumor to the axillary lymph nodes, indicating a potential link to aggressive tumor characteristics. To provide a comprehensive understanding, future studies should aim to elucidate the role of NDRG1 expression in different subcategories of Her2-negative tumors, including distinguishing between HR + and HR-subtypes. This would allow for a more refined conclusion regarding the implications of NDRG1 protein expression levels in breast cancer aggressiveness and prognosis.

### Electronic supplementary material

Below is the link to the electronic supplementary material.

Supplementary Material 1

Supplementary Material 2

Supplementary Material 3

## Data Availability

All data relating to the present study are available in this manuscript and supplementary files.
