# Supplementary material for: Association between NDRG1 protein expression and aggressive features of breast cancer: a systematic review and meta-analysis
Source: BMC Cancer. 2023 Oct 19;23:1003. doi: 10.1186/s12885-023-11517-7 (PMC10585795; doi:10.1186/s12885-023-11517-7)
Supplement: Supplementary file 1 — Supplementary Material 1 [file 12885_2023_11517_MOESM1_ESM.docx]

**Table S1. Search strategy**

**General search terms**

(NDRG1 OR RIT42 OR RIT42 OR “nickel-specific induction protein Cap43” OR “N-myc downstream regulated gene 1 protein” OR “N-myc downstream-regulated gene 1” OR “differentiation-related gene 1”) AND (Breast OR Mammary) AND (Neoplasm* OR Neoplasia* OR Tumor* OR Cancer* OR Carcinoma* OR Malignan*)

**PubMed**

**30 March 2023**

| **No.** | **Query** | **Results** |
| --- | --- | --- |
| 4 | #1 AND #2 AND #3 | 80 |
| 3 | (Cancer) OR (Cancer[MeSH Terms]) | 4,827,546 |
| 2 | (Breast) OR (Breast[MeSH Terms]) | 607,196 |
| 1 | ((NDRG1) OR (NDRG1[MeSH Terms])) OR (NDRG1[MeSH Terms]) | 704 |

**Scopus**

**30 March 2023**

| **No.** | **Query** | **Results** |
| --- | --- | --- |
| 3 | 1 AND 2 | 116 |
| 2 | TITLE-ABS-KEY ( breast OR mammary ) | 913,124 |
| 1 | TITLE-ABS-KEY ( ndrg1 OR rit42 OR rit42 OR "nickel-specific induction protein cap43" OR "n-myc downstream regulated gene 1 protein" OR "n-myc downstream-regulated gene 1" OR "differentiation-related gene 1" ) | 923 |

**MEDLINE**

| **No.** | **Search terms/Search strategy** | **Date** |
| --- | --- | --- |
| 1 | (NDRG1 OR RIT42 OR RIT42 OR “nickel-specific induction protein Cap43” OR “N-myc downstream regulated gene 1 protein” OR “N-myc downstream-regulated gene 1” OR “differentiation-related gene 1”) AND (Breast OR Mammary) AND (Neoplasm* OR Neoplasia* OR Tumor* OR Cancer* OR Carcinoma* OR Malignan*)  Search results: 93 | **30 March 2023** |

**Embase**

**30 March 2023**

| **No.** | **Search terms/Search strategy** | **Date** |
| --- | --- | --- |
| 1 | (NDRG1 OR RIT42 OR RIT42 OR ‘nickel-specific induction protein Cap43’ OR ‘N-myc downstream regulated gene 1 protein’ OR ‘N-myc downstream-regulated gene 1’ OR ‘differentiation-related gene 1’) AND (Breast OR Mammary) AND (Neoplasm* OR Neoplasia* OR Tumor* OR Cancer* OR Carcinoma* OR Malignan*)  Search results: 162 | 30 March 2023 |

**Ovid**

| **No.** | **Search terms/Search strategy** | **Date** |
| --- | --- | --- |
| 1 | (NDRG1 OR RIT42 OR RIT42 OR “nickel-specific induction protein Cap43” OR “N-myc downstream regulated gene 1 protein” OR “N-myc downstream-regulated gene 1” OR “differentiation-related gene 1”) AND (Breast OR Mammary) AND (Neoplasm* OR Neoplasia* OR Tumor* OR Cancer* OR Carcinoma* OR Malignan*)  Search results: 581 | **30 March 2023** |

**ProQuest**

| **No.** | **Search terms/Search strategy** | **Date** |
| --- | --- | --- |
| 1 | (NDRG1 OR RIT42 OR RIT42 OR “nickel-specific induction protein Cap43” OR “N-myc downstream regulated gene 1 protein” OR “N-myc downstream-regulated gene 1” OR “differentiation-related gene 1”) AND (Breast OR Mammary) AND (Neoplasm* OR Neoplasia* OR Tumor* OR Cancer* OR Carcinoma* OR Malignan*)  Search results: 330 | **30 March 2023** |
