# Supplementary material for: Association between NDRG1 protein expression and aggressive features of breast cancer: a systematic review and meta-analysis
Source: BMC Cancer. 2023 Oct 19;23:1003. doi: 10.1186/s12885-023-11517-7 (PMC10585795; doi:10.1186/s12885-023-11517-7)
Supplement: Supplementary file 3 — Supplementary Material 3 [file 12885_2023_11517_MOESM3_ESM.pdf]

# Association between NDRG1 protein expression and aggressive features of breast cancer: A systematic review and meta-analysis

Kwuntida Kotepui[1], Manas Kotepui[1*], Hideyuki J. Majima[1], Jitbanjong Tangpong[1]

[1]Medical Technology, School of Allied Health Sciences, Walailak University, Tha Sala, Nakhon Si Thammarat, Thailand

***Corresponding author**

Manas Kotepui: manas.ko@wu.ac.th

## Supplementary Figues

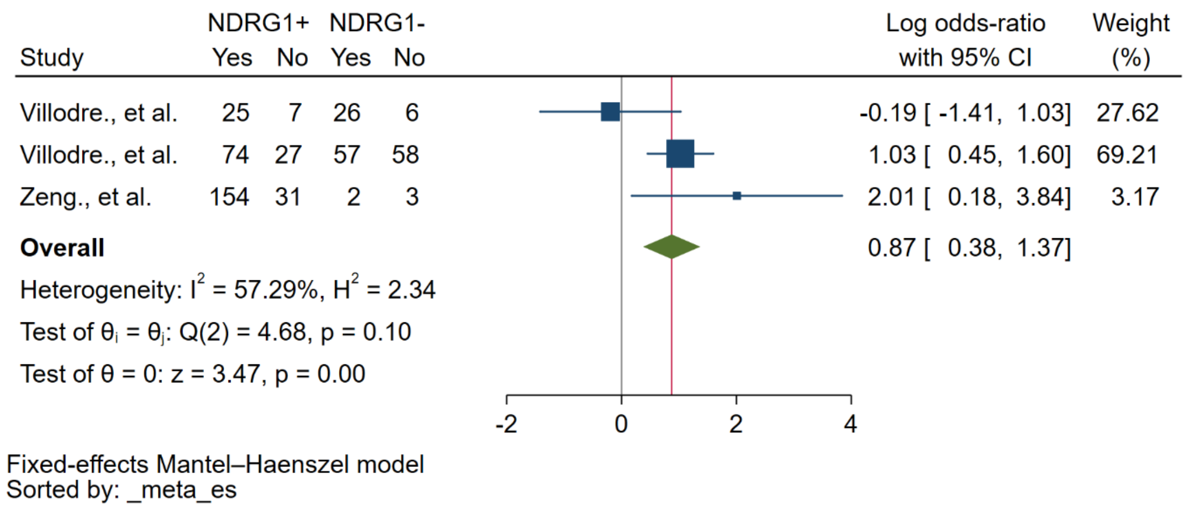


**Supplementary Figure 1.** The association between NDRG1 protein expression and aggressive features of breast cancer according to the fixed-effects model.

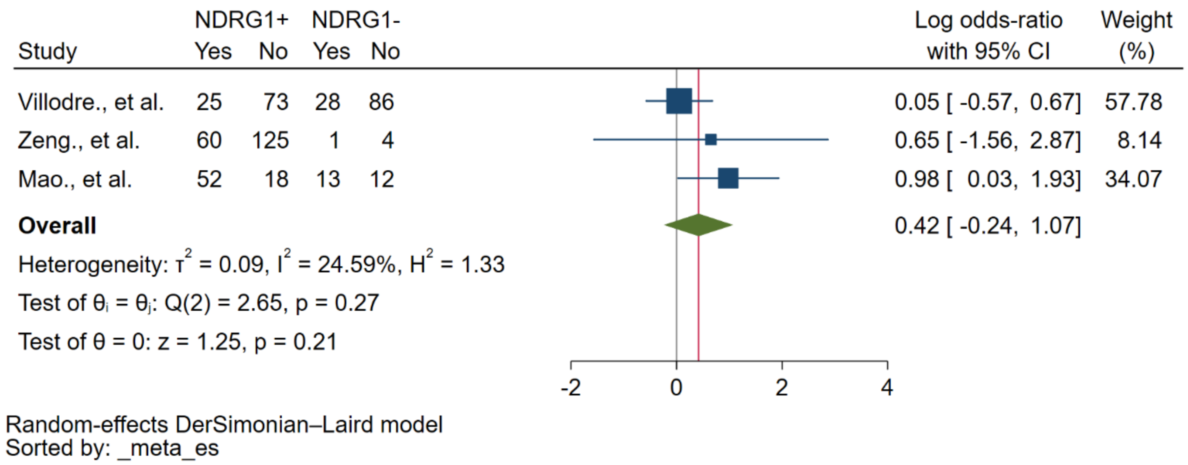


**Supplementary Figure 2.** The association between NDRG1 protein expression and tumor stage was identified according to the random effects model.

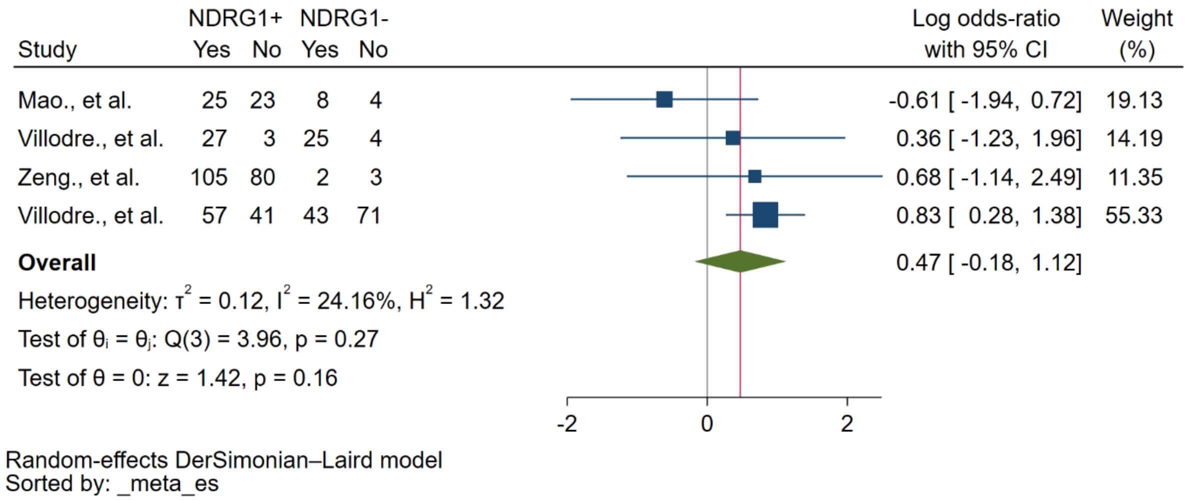


**Supplementary Figure 3.** The association between NDRG1 protein expression and axillary lymph node metastasis according to the random effects model.

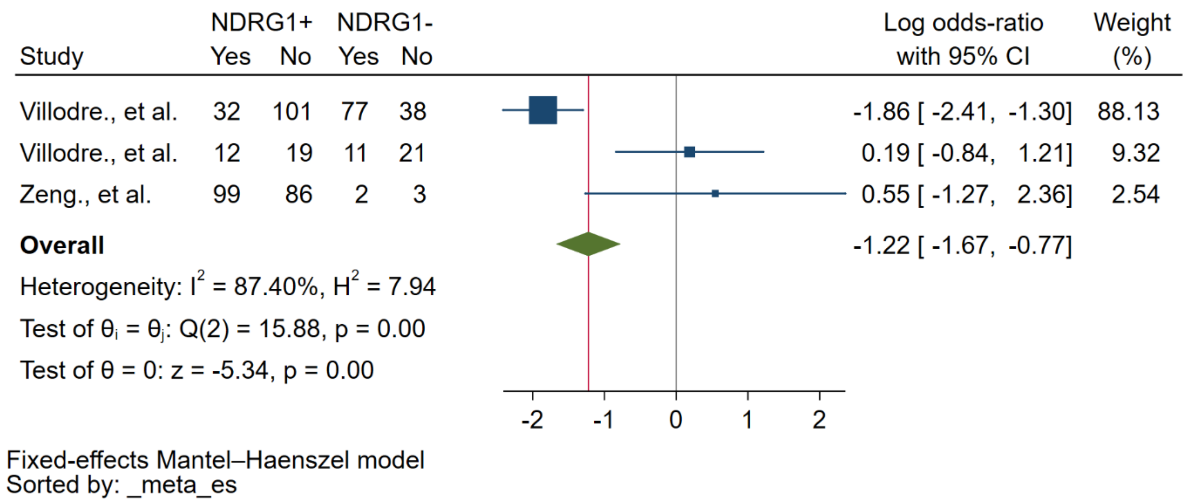


**Supplementary Figure 4.** The association between NDRG1 protein expression and ER status according to the random effects model.

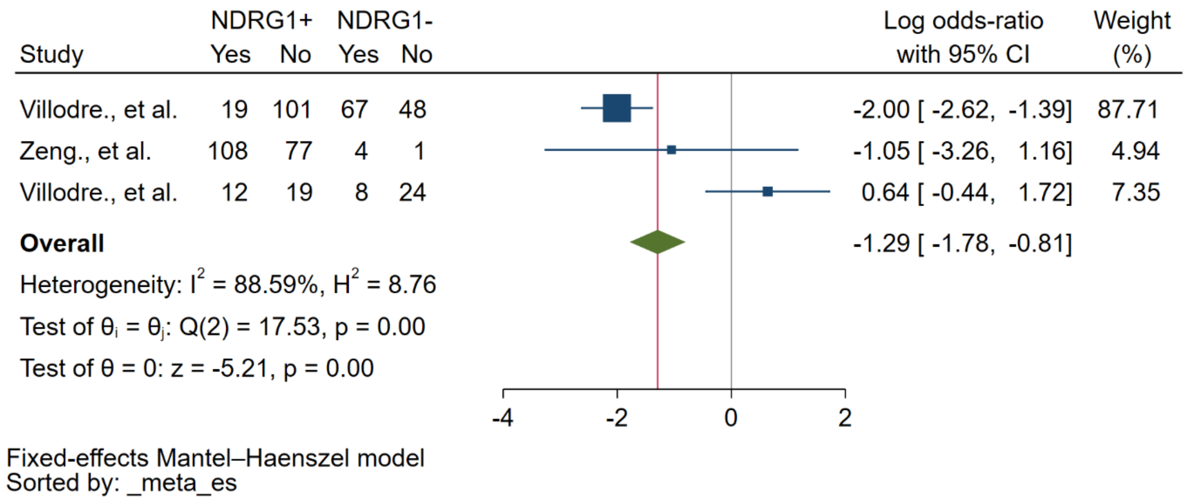


**Supplementary Figure 5.** The association between NDRG1 protein expression and PR status according to the fixed-effects model.

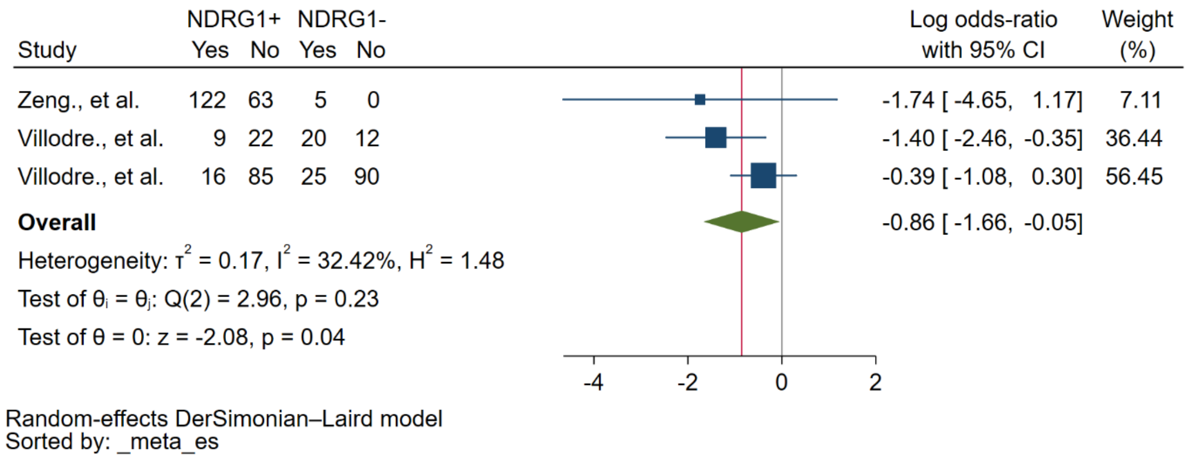


**Supplementary Figure 6.** The association between NDRG1 protein expression and Her2 status according to the random effects model.
